# Managing peatland vegetation for drinking water treatment

**DOI:** 10.1038/srep36751

**Published:** 2016-11-18

**Authors:** Jonathan P. Ritson, Michael Bell, Richard E. Brazier, Emilie Grand-Clement, Nigel J. D. Graham, Chris Freeman, David Smith, Michael R. Templeton, Joanna M. Clark

**Affiliations:** 1 Grantham Institute: Climate and Environment, Imperial College London, South Kensington, London SW7 2AZ, UK; 2 Department of Civil and Environmental Engineering, Imperial College London, South Kensington, London, SW7 2AZ, UK; 3 Department of Geography and Environmental Science; School of Archaeology, Geography and Environmental Science; University of Reading, Whiteknights, PO Box 227, Reading, RG6 6AB, UK; 4 Geography, College of Life and Environmental Sciences, University of Exeter, EX4 4RJ, UK; 5 Wolfson Carbon Capture Laboratory, School of Biological Sciences, Bangor University, Bangor, Gwynedd, LL57 2UW, UK; 6 South West Water, Peninsula House, Rydon Lane Exeter, Devon EX2 7HR, UK

## Abstract

Peatland ecosystem services include drinking water provision, flood mitigation, habitat provision and carbon sequestration. Dissolved organic carbon (DOC) removal is a key treatment process for the supply of potable water downstream from peat-dominated catchments. A transition from peat-forming *Sphagnum* moss to vascular plants has been observed in peatlands degraded by (a) land management, (b) atmospheric deposition and (c) climate change. Here within we show that the presence of vascular plants with higher annual above-ground biomass production leads to a seasonal addition of labile plant material into the peatland ecosystem as litter recalcitrance is lower. The net effect will be a smaller litter carbon pool due to higher rates of decomposition, and a greater seasonal pattern of DOC flux. Conventional water treatment involving coagulation-flocculation-sedimentation may be impeded by vascular plant-derived DOC. It has been shown that vascular plant-derived DOC is more difficult to remove via these methods than DOC derived from *Sphagnum*, whilst also being less susceptible to microbial mineralisation before reaching the treatment works. These results provide evidence that practices aimed at re-establishing *Sphagnum* moss on degraded peatlands could reduce costs and improve efficacy at water treatment works, offering an alternative to ‘end-of-pipe’ solutions through management of ecosystem service provision.

Organic rich peat soils are a major store of carbon worldwide, containing between 15–30% of the world’s total soil carbon^1^. Their existence is predicated on high year-round water-tables which create an anoxic environment, thus limiting decay, and also on the recalcitrance of plant litter (dead plant material) commonly found in peatland areas^2^. Dissolved organic carbon (DOC) is a significant flux of carbon from peatlands^3^ with implications for both carbon budgets and potable water treatment. DOC concentrations have been rising in many parts of Europe and North America due to warming, drought induced enzymatic activity, increased atmospheric CO_2_ concentrations and recovery from catchment sulfate driven-acidification^4^,^5^,^6^,^7^.

DOC affects water treatment by increasing coagulant demand and thus sludge production, reducing disinfectant efficacy and/or increasing disinfectant demand as well as providing a substrate for microbial regrowth^8^. It can also lead to colour, odour and taste problems and so must be removed during treatment, conventionally by coagulation, flocculation and sedimentation/flotation. DOC which remains post-coagulation can react with chemical disinfectants to form disinfection by-products (DBPs)^9^, some of which are regulated due to associated human health concerns including genotoxicity and carcinogenicity^10^. DOC in the UK has been shown to become increasingly difficult to treat via coagulation, suggesting ever higher treatment costs or the need for new treatment processes in the future^11^.

Catchment management represents a radical alternative to current end-of-pipe treatment as a response to rising DOC and a number of peatland restoration programmes, such as the Exmoor Mires project in south-west England, have aimed to promote high water-tables for *Sphagnum* dominance, peat stability, reduced flood risk and drinking water provision^12^. Schemes such as these offer a way of positively managing the ecosystem services provided by peatlands, however further evidence is needed of their efficacy. The vegetative source of DOC is significant as radiocarbon studies have suggested that the majority of DOC in surface waters has recently entered the system and therefore most likely originates from decaying litter and the upper soil horizons^13^,^14^. Vegetative change in peatlands may occur due to climate change, land management and nitrogen deposition, with species dominance shifting from *Sphagnum* mosses to vascular plants^15^ and many typically grassland vascular species (*Juncus effusus, Molinia caerulea*) are colonising peatland areas^16^,^17^,^18^.

The present research has sought to quantify the effect of changing litter input in peatlands on DOC, considering both typical peat-forming species (*Calluna* and *Sphagnum*) as well as encroaching grassland species (*Juncus* and *Molinia*). We consider how effective water treatment processes are for different DOC sources, the persistence of this organic carbon as well as how the different vegetation litters decompose, both in laboratory and field experiments, and the implication this will have for C retention in peatlands and downstream water treatment works. This allows us to test the hypothesis that catchment management may offer an alternative to end-of-pipe solutions at water treatment works.

## Results

### Ease of DOC removal during the treatment process for different peatland sources

First, we evaluated how easy it was to treat DOC from different vegetation types using conventional methods. Samples of DOC extracted from *Sphagnum, Calluna, Juncus, Molinia* and a peat soil, sourced from Exmoor National Park (51°07′23.3″N 3°45′11.8″W) were subject to coagulation/flocculation ‘jar testing’ with ferric sulfate, a practice used in the water industry to simulate DOC removal at water treatment works. Optimal pH and coagulant dose were found for each DOC source (see Supplementary Figures 1 and 2) and then post-coagulation DOC was disinfected with chlorine to assess the potential to form DBPs. Fluorescence and ultra-violet spectroscopy were used to determine which factors control the removal of DOC as these techniques have shown capability as sensors for online management of water treatment processes.

**Figure 1:**
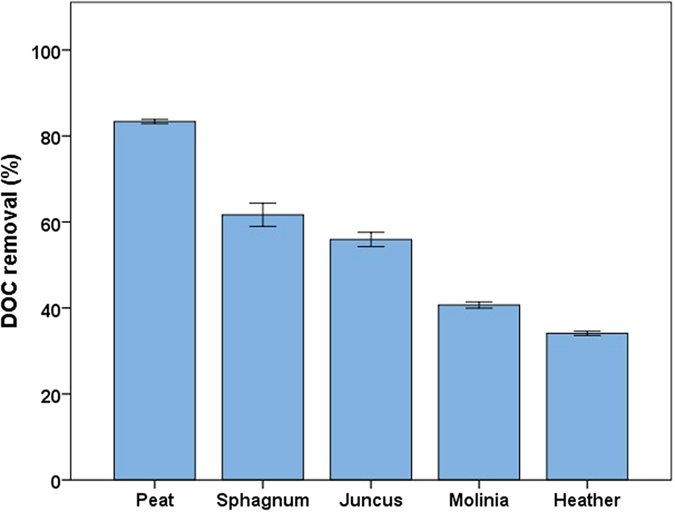
DOC removal by coagulation for different peatland sources. Error bars at one standard error (n = 3).

The peat soil and *Sphagnum* litter, which are currently encouraged by catchment management for biodiversity and C sequestration reasons, gave the most effective removal by coagulation with 83.4 ± 0.4 and 64.1 ± 2.0%, respectively (see Fig. 1). These were followed by the vascular plants with removal in the order *Juncus* (55.9 ± 1.4%), *Molinia* (40.7 ± 0.6%) and then *Calluna* (34.1 ± 0.4%). The source of the DOC has a significant effect (ANOVA *p* < 0.001) on DOC removal with a Tukey HSD test suggesting the sources belonged to statistical subsets of peat > *Sphagnum* and *Juncus *> *Molinia *> *Calluna*. The ratio of ‘humic-like’ fluorescence peak C to ‘protein-like’ peak T showed a strong correlation with removal by coagulation (Spearman’s *ρ* = 0.971, *p* value <0.001). This would suggest that the humic-like compounds are readily removed whereas proteinaceous DOC is more difficult to remove from drinking water.

The coagulated samples were then disinfected by chlorination to assess their potential to form chloroform, a disinfection by-product which is regulated in many countries. *Calluna* had the highest potential to form chloroform (165.0 ± 6.9 μg mg^−1^) followed by *Sphagnum* (75.9 ± 4.3 μg mg^−1^), peat (66.7 ± 3.5 μg mg^−1^), *Juncus* (61.8 ± 1.6 μg mg^−1^) and then *Molinia* (50.1 ± 2.3 μg mg^−1^). The source of DOC had a significant effect on chloroform formation (ANOVA *p *< 0.001) with a Tukey HSD test suggesting the sources belonged to statistical subsets of *Calluna *> Sphagnum, peat and *Juncus *> *Molinia*.

The specific ultra-violet absorbance at 254 nm (SUVA) value, indicating aromaticity, correlated well with the decrease in DBP formation between pre- and post-coagulation samples (Spearman’s ρ = 0.949, p value < 0.001).

These results indicate catchment management which encourages *Sphagnum* dominance and peat formation will produce DOC which is easier to remove for the production of potable water than that arising from vascular plants, which also has comparable formation of chloroform. The encroachment of vascular plants into *Sphagnum* bogs introduce DOC sources which are poorly removed by coagulation and therefore may require alternative, more costly, treatment processes. The ease of removal appears to be controlled by the ratio of humic-like DOC to that which is more proteinaceous in character, demonstrated using fluorescence spectroscopy. The high formation of chloroform from *Calluna* is of concern as this is the dominant vegetation in many European heathlands and is actively encouraged in the UK for sport shooting habitat^19^.

### Persistence of DOC from peatland sources within the catchment

Next, we evaluated how DOC released from different sources could be degraded as it is transported through the peatland, into surface waters and finally to the water treatment works. Soil organic horizons can retain DOC produced from litter^20^ and formation of soil organic carbon can occur through microbial and physical pathways^21^. Recent work has suggested that lakes with long residence times have the ability to act as a buffer to changing DOC, partly negating increases in concentration and colour by mineralising carbon through microbial and photolytic routes^22^. This gives rise to the idea of a ‘river as a chemostat’, which suggests that as river order increases, variation in DOC quantity and quality decreases^23^.

**Figure 2:**
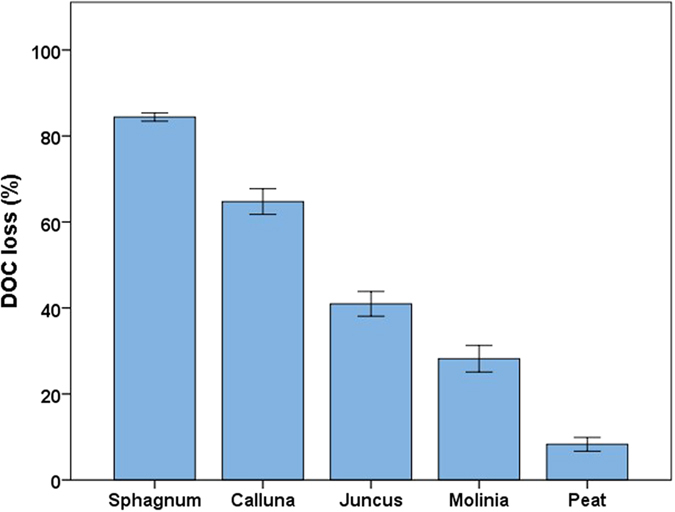
% loss of DOC on seven day incubation with added nutrients and standardised inoculum for different DOC sources. Error bars at one standard error (n = 5).

Seven-day incubations were performed on DOC extracted from *Sphagnum, Calluna, Juncus, Molinia* and a peat soil which monitored how much C was mineralised over the period, giving the biodegradable fraction or BDOC. The results suggest a significant effect of source on loss of DOC (ANOVA F = 147.92, p < 0.001, ω^2^ = 0.959) with all sources having different means at the p < 0.01 level, except *Juncus*-*Molinia* which were significantly different at the p < 0.05 level. Mean values for loss of DOC were in the order S*phagnum *> *Calluna *> *Juncus *> *Molinia *> peat (Fig. 2).

The SUVA is often used as a measure of aromaticity of DOC^24^. In this case the indicator of aromaticity correlated well with the loss of carbon during the incubation (Spearman’s ρ =  −0.978, p < 0.001, see Supplementary Figure 3) as reported elsewhere^25^. The E2:E4 ratio is also derived from UV data and has been used as an indicator of humification of DOC whereas the E2:E3 index is used as a proxy for DOC molecular weight^26^. These indices showed a significant increase in humification and molecular weight post-incubation whilst the aromaticity also increased (Wilcoxon signed rank test p < 0.05). This was true of all sources and parameters except peat DOC molecular weight which remained the same. This would suggest the preferential mineralisation of low molecular weight and aliphatic compounds, although the formation of aromatic compounds by bacteria may also occur^27^.

The microbial mineralisation resulted in greater homogeneity between sources. A large decrease in effect size was noted in pre- and post-incubation ANOVA tests with the ω^2^ estimate of effect size falling from 0.936 to 0.545 for the effect of source on DOC molecular weight. *Juncus* and *Molinia* still had significantly lower humification than the peatland sources post-incubation (p < 0.05) indicating chemical differences between DOC quality from grassland and peatland litter sources are persistent. Post-incubation, the aromaticity of the vascular plant DOC was statistically similar whereas *Sphagnum* and peat had higher values (p < 0.05 see Supplementary Figure 4).

As there were significant differences between DOC from the encroaching grassland sources (*Molinia* and *Juncus*) and the typical peatland sources (*Sphagnum, Calluna* and peat soil) after the incubations, differences in humification and molecular weight of DOC may persist within the catchment. *Sphagnum* has been shown to produce DOC which is highly labile, resulting in low DOC concentrations with a high level of aromaticity after microbial processing. This may be seen as positive from a water treatment perspective as much of the DOC from *Sphagnum* may be mineralised before it reaches the treatment works and that which remains is likely to be easily removed via coagulation/flocculation due to its high aromaticity^28^.

DOC arriving at the treatment works will be a mixture of allochthonous and autochthonous sources. Our results show that there are large differences between the major allochthonous sources in the catchment (coagulation experiment) and that these differences are persistent (BDOC experiment). The exact composition of DOC which will reach the treatment works will likely be somewhere in between these two ‘end-members’ of the fresh vegetation extracts and the incubated samples due to both processes within the peatland and during transport in surface waters. The relative contributions of the different catchment sources remains an area for further research.

### Litter decomposition in the laboratory

**Figure 3:**
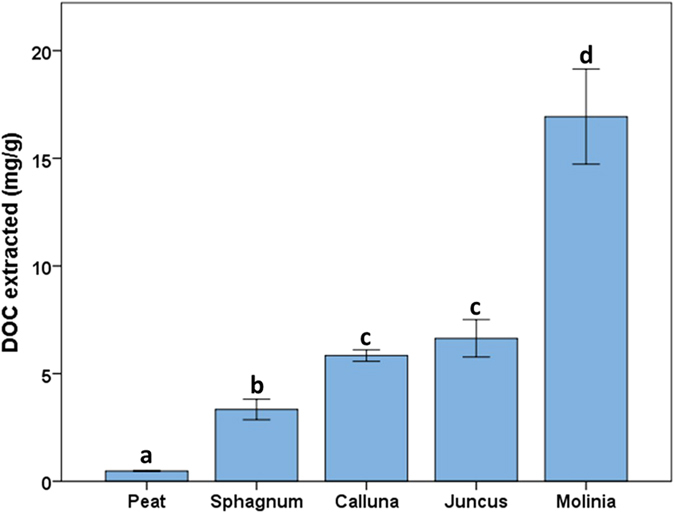
DOC production from different peatland sources (letters indicate statistical subsets). Error bars at one standard error (n = 5).

Subsequently, we examined how the starting chemistry of vegetation litter controls the amount of DOC produced during laboratory decomposition. A six-week simulation was undertaken using vegetation samples (*Sphagnum, Calluna, Juncus, Molinia*) and a peat soil which decomposed in a climate control chamber with regular simulated rainfall events using reverse osmosis water. Sub-samples were analysed for carbon and nitrogen content and at the end of the experiment DOC was extracted from the samples. The amount of extractable DOC is shown in Fig. 3, with the invasive species *Molinia* producing significantly more than the typical peatland sources of DOC.

The ratio of carbon to nitrogen is often used as a measure of nutrient availability or the decomposability of the vegetation with high values indicating poor nutrient availability. Analysis on the starting material showed C:N ratios to be in the observational order *Sphagnum *> *Calluna *> *Juncus *> *Molinia *> peat (see Supplementary Table 1). Correlations between litter C:N ratios and water extractable DOC have been reported elsewhere in the literature^29^ and were again found to correlate strongly (Spearman’s ρ = −0.884, p < 0.001). Although the C:N content of soils has been shown to be a predictor of riverine DOC concentration^30^, including the peat soil in this dataset gave poor results (Spearman’s ρ = 0.043, p = 0.838). The peat soil had the highest %N which, given that the peat soil is composed of the decaying vegetation above it, suggests preferential preservation of nitrogen containing compounds in peat, which has been noted elsewhere^31^. The ratio is therefore measuring the extent of N protection rather than potential DOC flux, hence the lack of a correlation when including both litter and soils in the dataset.

In an additional experiment, *Calluna* flowers and twigs were separated and analysed for C and N concentrations before DOC extraction and analysis. Elemental analysis showed a C:N ratio of 49.41 ± 0.40 for the flowers compared to 65.83 ± 0.34 for the twigs. The flowers were found to produce 19.8% more DOC on a mg g^−1^ basis (t = 3.107, p = 0.021) and this DOC was of a higher aromaticity, indicated by the SUVA value (t = 15.824, p < 0.001). These results would suggest that the flowers provide a seasonal addition of more labile material (low C:N) which is likely to decompose quickly and which has a greater potential to produce DOC. The DOC produced is of a more aromatic character and so, from the BDOC experiment, is likely to be more persistent in surface waters.

The litter C:N values and correlation with DOC production would suggest vascular plants are more able to utilise nitrogen, resulting in higher nutrient availability to decomposers and a corresponding higher amount of DOC production.

### Litter decomposition in the field

The scaling-up of laboratory findings of DOC flux to catchment scale is often complex and the dominant driver can change when moving from laboratory to the field^32^. An experiment using ‘litterbags’, mesh bags which can be buried in the field to experience realistic decay conditions, was performed, in part to bridge this gap from lab to field. A series of sites near Postbridge in Dartmoor National Park, UK (50°6′N; 3°90′W) were selected and instrumented with temperature and water-table monitoring (see Methods for site details and climate data).

The litterbags were retrieved after ten months of decomposition between December and September. These were then weighed for mass loss, DOC was extracted, and then the resulting data were analysed in an ANCOVA model using mean temperature and depth to water table as covariates. Only the mass loss of samples will be discussed as only very small effects on DOC quality were noted (see Supplementary Table 2).

Mass loss during decomposition was in the order *Calluna* (33.6 ± 2.9%), *Juncus* (24.5 ± 1.2%), *Molinia* (21.3 ± 1.3%) then *Sphagnum* (1.2 ± 0.3%). All vegetation types had significantly different mass loss at the p = 0.01 level except the *Juncus*-*Molinia* comparison, which were statistically similar. The very small mass loss of *Sphagnum* suggests that although low DOC production was observed in the laboratory decomposition experiment on a mg per g basis, a large carbon pool can build up over many years, potentially meaning high overall flux on a gC m^−2^ year^−1^ basis. The vascular plants, in contrast, showed high DOC production on a mg per g basis in the laboratory decomposition yet showed very rapid mass loss in the field, suggesting the overall litter carbon pool in a vascular plant dominated system will comprise just a few years’ worth of litter. This could have important implications for DOC flux from catchments where grassland species are encroaching on *Sphagnum* peatlands^33^ as higher peaks in flux may be observed from vascular plant biomass as it rapidly decays, however as the size of the carbon pool is likely to decrease, DOC flux may reduce overall.

### Seasonality of DOC quantity, quality and treatability due to vegetation source

There are large differences reported in the literature for both litter production rates of the different plant species studied and the timing of their litter production, as shown in Table 1.

**Figure 4:**
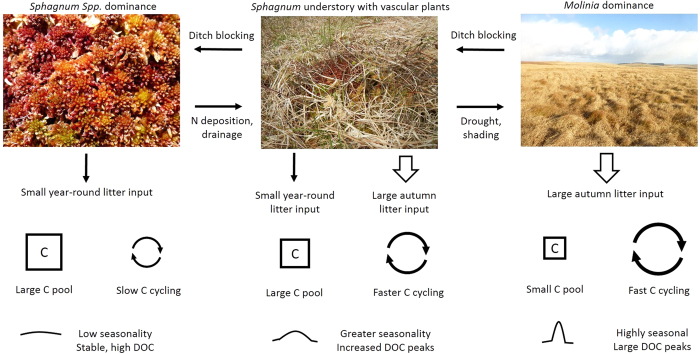
Conceptual diagram showing changes to size, speed of cycling and seasonality of litter carbon pool on transition from *Sphagnum* to *Molina* domination of uplands.

The literature values of litter production suggest very large annual input from *Juncus* and *Molinia* with smaller inputs from *Calluna* and *Sphagnum. Sphagnum* and *Calluna* both produce litter year-round (although *Calluna* peaks in October) whereas *Molinia* and *Juncus* only produce litter in late autumn. A large part of the autumn/winter peak in *Calluna* litter production occurs as the flower capsules fall^34^. As we showed in the previous experiments, this adds a large amount of labile material to the litter layer, resulting in greater DOC production. This suggests that a shift from *Sphagnum* to *Calluna, Molinia* or *Juncus* dominated areas could lead to a stronger seasonal signal in DOC fluxes from the litter layer. This concept of increased seasonality and altered sizes and cycling of litter carbon pools is summarised in Fig. 4. This conceptual diagram offers new insight into likely changes in carbon cycling in peatlands due to vegetation changes caused by atmospheric pollution, land management and climate change. This is likely to add further complexity to the number of cycles already affecting DOC production and transport, many of which operate at different scales and temporal cycles^35^.

The role of vegetation in controlling seasonality of soil and riverine DOC concentrations has not been well studied (see Supplementary Table 3), although field evidence for this conceptual framework exists. A study in southern Norway showed at 0–10 cm in depth, *Molinia* and *Calluna* gave higher soil pore-water DOC concentrations than *Sphagnum* with a strong seasonal component, timed with litter production. At 10–20 cm in depth, however, *Sphagnum* had higher DOC concentrations than the vascular plants due to an increased C pool and this was stable throughout the year^36^. These field data add weight to this new conceptual understanding of how changing peatland vegetation may alter C cycling in peatlands leading to larger, seasonal peaks in DOC. Further work in the UK has suggested that DOC in porewater beneath *Sphagnum* is higher than *Molinia*^37^, however seasonality and depth were not considered in this work and a subsequent study by the authors showed no effect of plant functional type on DOC concentrations only ecosystem respiration, with a notable seasonal component from vascular plants^38^. Further field evidence is needed to account for the differences found between these field studies.

The potential effect of more seasonal DOC export on peatland organic matter decomposition remains an area for future research, as is the role of below-ground biomass and the potential priming effect of root exudates. This could be significant as, for example, *Molinia* produces ten-times the annual root biomass of *Calluna*^39^ and root exudation can destabilise peat organic matter by adding labile carbon which ‘primes’ the decomposition of peat^15^,^40^. Vegetation type also affects the microbial community diversity in peatlands^41^ which may have implications for carbon sequestration and DOC flux.

The value of the water regulation ecosystem service performed by blanket bogs in the UK has been estimated at £230 million per year^42^, however there are multiple co-benefits to catchment management, including flood attenuation, carbon sequestration, biodiversity, agriculture and amenity value^12^,^43^ which may improve the economics of such schemes. The importance of vegetation type for litter layer DOC flux has been shown and highlights the need to include *Sphagnum* in models considering DOC flux at the catchment scale as recent work, which used remote sensing which could not discriminate between *Sphagnum* and vascular plants, have shown smaller effects^44^.

Our research provides important early evidence that restoring *Sphagnum* moss cover may deliver improved drinking water quality and indicates potential for a payments for ecosystem services (PES) model whereby land managers could be paid for activities like ditch blocking for drinking water provision^43^. Current catchment management schemes have used ditch blocking to raise water-tables in peatland areas to provide conditions favorable for *Sphagnum*, peat formation and water retention with variable successes^12^. Preliminary results from the Exmoor Mires project have suggested this has caused a decrease in overall DOC load downstream due to lower peak flows^45^ and an increase in species adapted to wetter conditions^46^. Our data provide the first compelling evidence that catchment management approaches offer benefits in relation to water treatment process with lower seasonal DOC peaks and improved treatability, although this must be tested in scaling up to the catchment level. These results show that changes in vegetation can have a large effect on the seasonality and treatability of DOC in peatland catchments and therefore managing vegetation type could be a method of improving raw water quality rather than ‘end-of-pipe’ solutions at the treatment works.

## Methods

### Site details and litter collection

Field sites near current catchment management scheme were selected in both Exmoor and Dartmoor National Parks. For vascular plants, litter was collected as standing dead biomass. For *Sphagnum*, whole sods were collected for processing in the laboratory. Peat samples were collected using a screw augur, and peat from 10–30 cm depth was used in the experiments. As the decomposition of *Sphagnum spp*. is a continuum process, the section 2–4 cm below the tip of the capitulum was taken as representative of freshly senesced “litter”, in line with other studies^47^.

Samples for the coagulation optimisation and laboratory decomposition experiment were collected from two sites in Exmoor National Park, UK; Aclands (51°07′54.2″N 3°48′43.3″W) at approximately 450 m elevation and Spooners (51°07′23.3″N 3°45′11.8″W) at approximately 400 m elevation. The National Park contains a mixture of blanket mire, wet and dry heath, grassland and scrub communities, whereas the specific sites are bogs dominated by *Molinia caerulea, Sphagnum Spp., Juncus effusus, Calluna vulgaris and Eriophorum vaginatum.* Samples of peat taken were approximately H7 on the Von Post scale, indicating dark, well humified peat with only a few recognisable plant structures remaining.

Seven instrumented sites across an altitudinal gradient were set up near Postbridge (50°6′N; 3°90′W) in Dartmoor National Park in 2014 by Michael Bell of University of Reading. Peat samples collected from these sites were approximately H6 on the Von Post scale suggesting reasonably well humified material with some plant structures remaining. The sites contain a mixture of *Molinia caerulea, Sphagnum spp., Juncus effusus, Calluna vulgaris, Erica tetralix, Eriophorum vaginatum, Trichophorum cespitosum and Drosera rotundifolia*.

### Litterbag experiments

Litterbags constructed from polyamide monofil were purchased from Filtrations Technik (Germany) in 10 × 10 cm size with a 0.5 mm mesh. The litterbags were installed across an altitudinal gradient with site 2 at an elevation of 365 m above sea level, site 5 at 503 m and site 7 at 528 m. Temperature and water table depth were recorded at 15 minute intervals. See Supplementary Table 4 for details. The range of depth to water table at each suggest the litterbags experienced some time both above and below the water table, thus being representative of the conditions of recently senesced litter.

Vegetation collected from the Dartmoor sites was used for the litterbag experiment using the methodology detailed above. The vegetation was air-dried to a constant weight and then cut to 2 cm length and homogenised. Litterbags were filled with approximately 3 g vegetation except *Sphagnum* as 1 g was sufficient to fill the litterbag due to its low density. The bags were sealed by double stitching with Nylon thread.

Five replicates of each vegetation type were installed at approximately 10 cm depth, below the actively growing vegetation and so thus in the litter layer. After ten months the litterbags were collected and analysed for mass loss and water extractable DOC. To assess the impact of the flowers of *Calluna vulgaris* on DOC flux from litter, samples were collected in August 2015. Samples were separated into the flower capsules and small twigs. After separation the samples were homogenised and extracted for DOC which was then analysed for UV and fluorescence properties. A subsample was analysed in duplicate for C and N content.

### Laboratory decomposition experiment

Approximately 2 g dry-weight of air-dried vegetation/peat from the Exmoor sites were placed in 7 cm Buchner funnels fitted into amber-glass bottles used to collect the simulated rainfall. A lower weight of sample was used for *Sphagnum* (~0.65 g) and *Molinia* (~1.5 g) due to their low density when air-dried, meaning this was enough vegetation to fill the Buchner funnel. The peat samples were pressed lightly into the funnel so that a seal was created, meaning rainfall had to infiltrate the peat rather than bypass through the funnel.

Five sub-samples of each vegetation type were oven-dried at 70 °C until constant weight, to determine the air-dry to oven-dry conversion factor. These sub-samples were then ground to the consistency of flour using a disc mill (Tema, UK) and analysed for carbon and nitrogen content.

Data for the months of June, July and August from the regional historic climate records of the UK Meteorological Office for the south west of England for the period 1910–2013 (310 months in total from the record) were used to find the 50^th^ percentile for total monthly rainfall. This value, 79.0 mm, was used as representative of normal summer rainfall conditions and was applied to the samples over the incubation. Temperature cycled between twelve hours at the mean daily maximum of 18.9 and the mean daily minimum of 10.7 °C based on the same historical datasets for the south west region.

The samples were then placed in an incubator for six weeks with rainfall events occurring eleven times per month (regional average for June, July and August), applied drop-wise over the area of the funnel with a graduated syringe. Reverse osmosis (RO) treated water has been used in similar degradation studies and is employed so that no organic carbon is added to the samples. As the samples were collected from the field and had been in contact with litter and soil no inoculation with microorganisms was required as a suitable decomposer community was likely to be present. At the end of the six week simulation the samples were air-dried and weighed. DOC was then extracted from the air dried samples.

### Laboratory procedures

DOC was extracted from soil and vegetation samples using approximately 20:1 ratio of RO treated water to sample. Samples were filtered using pre-combusted GF/F filters (Whatman, UK), acidified to ~pH 2 using HCl and stored at 4 °C before analysis. DOC was measured as non-purgeable organic carbon (NPOC) on a Shimadzu TOC-V instrument which uses a UV/persulphate oxidation method. The method detection limit was found to be 0.05 mg l^−1^. UV absorbance was measured on a Perkin Elma Lambda 3 using a 1-cm pathlength quartz cuvette and the specific absorbance, SUVA, was calculated as the absorbance at 254 nm in units of m^−1^ divided by the NPOC content (mgC l^−1^).

Elemental analysis was performed in duplicate on a Thermoflash C:N analyser. Aspartic acid (Sigma Aldrich) was used as a reference material for the overall performance of the instrument and standard material for soil and vegetation (provided by University of Reading) was also used to test instrument performance in more complicated matrices. The BDOC fraction was determined as recommended in a review of methods^48^ by determining loss of DOC after a seven day incubation with added nutrients. The average standard error of replicates from 25 samples was 1.49% and a dextrose control (to check viability of the microbial community) gave 96.3% DOC removal.

Although many classes of DBPs are formed during water treatment this study focusses on chloroform, a trihalomethane (THM), as this class of compounds are regulated in the UK and chloroform was consistently detected in all samples. Brominated forms were not detected due to the use of RO water. The chlorination procedure followed that of other studies^49^ using US EPA method 551.1 for quantification on a Shimadzu gas chromatograpy system with electron capture detection (GC-ECD). All chromatogram peaks were normalised to that of an internal standard, bromo-fluoro-benzene (Sigma Aldrich), added to each sample. A method detection limit of 0.4 μg l^−1^ was achieved.

For the coagulation experiment a range of dosages and pH were tested using the methods found in US EPA ‘Enhanced Coagulation and Enhanced Precipitative Softening Guidance Manual’ as well as ASTM method 2035-08 as a guide. This involved finding the ‘point of diminishing return’, the point at which adding a further 10 mg l^−1^ coagulant removes less than 0.3 mg l^−1^ DOC. These dosages were then used to test pH in the range 4.5–6.0 based on literature reports of effective removal for ferric sulphate^50^. All jar testing was performed on a Phipps and Bird PB-700 paddled jar-tester (Phipps and Bird Ltd., Virginia, USA) and after settling, filtered through Whatman qualitative grade #2 filters to remove the remaining flocs before measurement of DOC. Ferric sulphate was used as the coagulant with calcium hydroxide to stabilise pH. Coagulation conditions involved a rapid mix at 100 rpm for one minute, a slower mix at 30 rpm for 30 minutes, followed by settling for one hour. Initial dosage and pH tests were performed in duplicate. Final tests were performed in triplicate.

For fluorescence analysis, samples were scanned at excitation wavelengths between 220 and 450 nm at 5 nm intervals and the resulting emission observed between 300 and 600 nm at 2 nm intervals using a Vary Eclipse fluorescence spectrophotometer. An R script was developed based on published scripts^51^ to process the data and normalise to the Raman scattering peak of RO water. Fluorescence excitation-emission matrices (EEM) is commonly split into a number of fluorphores which are known to emit at certain wavelengths. The ‘peak C’ measure, related to humic-like character, was defined as the maximum intensity of emission between 380 and 460 nm from excitation between 300 and 360. The tryptophan-like peak, ‘peak T’, suggestive of protein-like character, was defined as the maximum intensity of emission between 320–360 nm from excitation between 270–290 nm.

## Additional Information

**How to cite this article**: Ritson, J. P. *et al*. Managing peatland vegetation for drinking water treatment. *Sci. Rep.* **6**, 36751; doi: 10.1038/srep36751 (2016).

**Publisher’s note:** Springer Nature remains neutral with regard to jurisdictional claims in published maps and institutional affiliations.

## Supplementary Material

Supplementary Information

## Figures and Tables

**Table 1:** Literature estimates of amount and seasonality of litter production for the species of interest.

Vegetation	Litter production (g m^−2^ year ^−1^)	Timing	References
*Sphagnum spp.*	35–156	Year-round	52
*Calluna vulgaris*	40–261	Year-round, peaks in autumn/winter	34,53
*Juncus effusus*	690–800	Sept–Nov	54
*Molinia caerulea*	536–633	Sept–Nov	55
