# Supplementary material for: Managing peatland vegetation for drinking water treatment
Source: Sci Rep. 2016 Nov 18;6:36751. doi: 10.1038/srep36751 (PMC5114669; doi:10.1038/srep36751)
Supplement: Supplementary Information [file srep36751-s1.pdf]

Managing peatland vegetation for drinking water treatment

Authors: Jonathan P. Ritson[1,2,3*], Michael Bell[3], Richard E. Brazier[4], Emilie Grand-Clement[4], Nigel J.D. Graham[2], Chris Freeman[5], David Smith[6], Michael R. Templeton[2] and Joanna M. Clark[3].

[1] Grantham Institute: Climate and Environment, Imperial College London, South Kensington, London SW7 2AZ, UK

[2] Department of Civil and Environmental Engineering, Imperial College London, South Kensington, London, SW7 2AZ, UK

[3] Department of Geography and Environmental Science; School of Archaeology, Geography and Environmental Science; University of Reading, Whiteknights, PO Box 227, Reading, RG6 6AB, UK

[4]Geography, College of Life and Environmental Sciences, University of Exeter, EX4 4RJ, UK

[5] Wolfson Carbon Capture Laboratory, School of Biological Sciences, Bangor University, Bangor, Gwynedd, LL57 2UW, UK

[6]South West Water, Peninsula House, Rydon Lane Exeter, Devon EX2 7HR

*Corresponding author (j.ritson12@imperial.ac.uk; j.p.ritson@reading.ac.uk)

Supplementary information

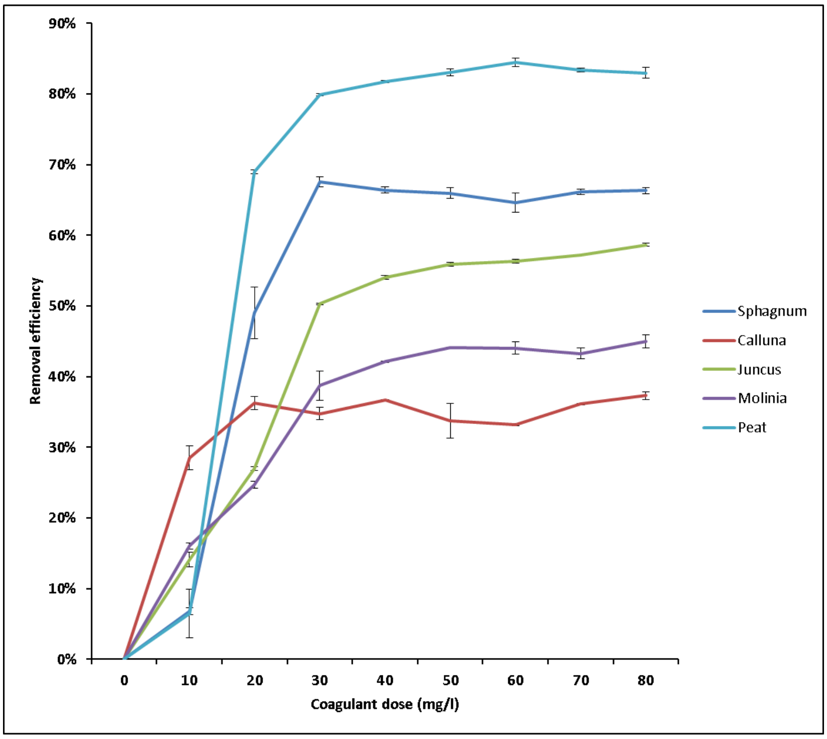

Supplementary Figure 1: Dose optimisation for DOC sources for coagulation with ferric sulphate. Error bars at one standard error.

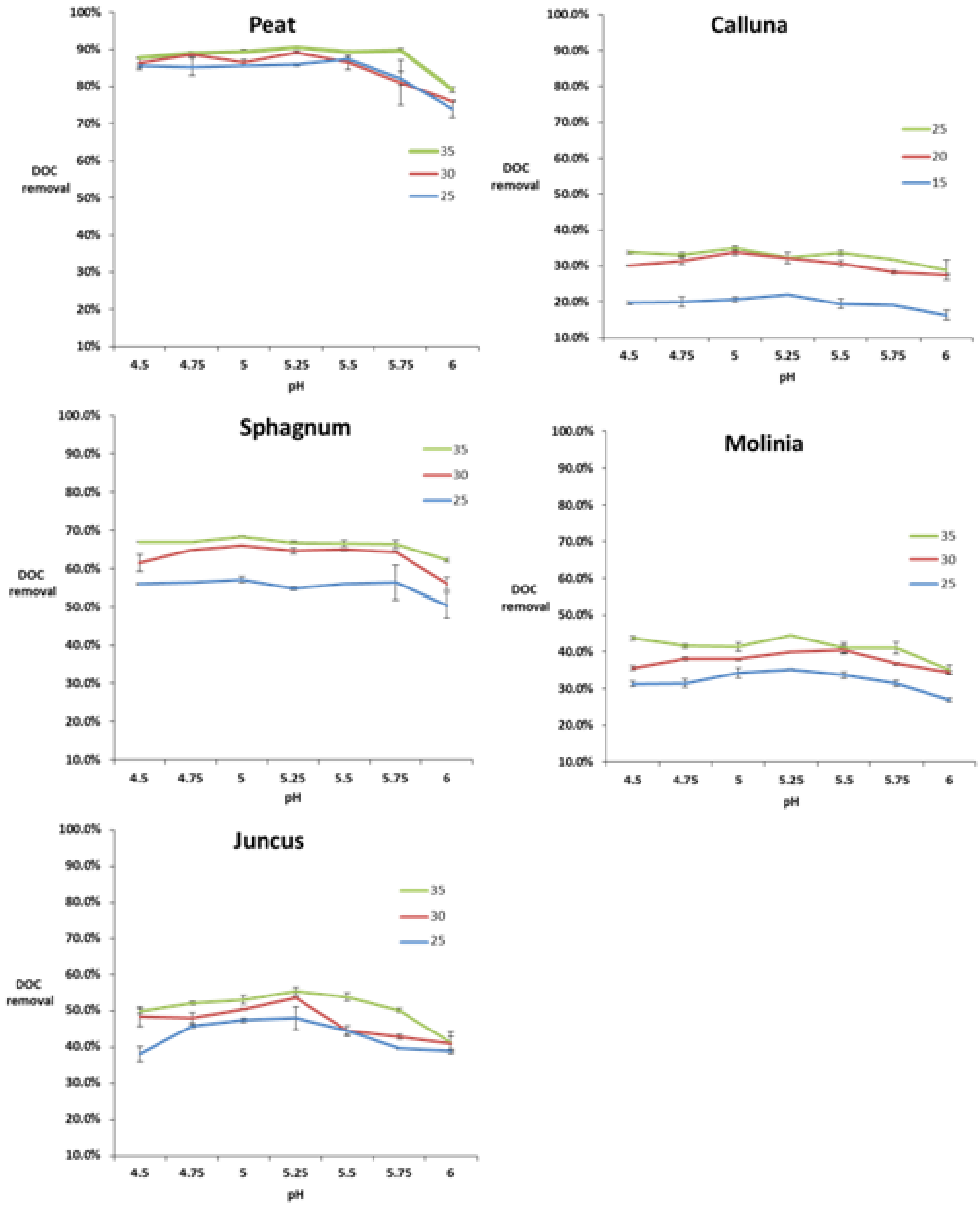

Supplementary Figure 2: pH optimisation for DOC sources for coagulation with ferric sulphate at 'point of diminishing returns' dosage ±5 mg l^{-1}. Error bars at one standard error.

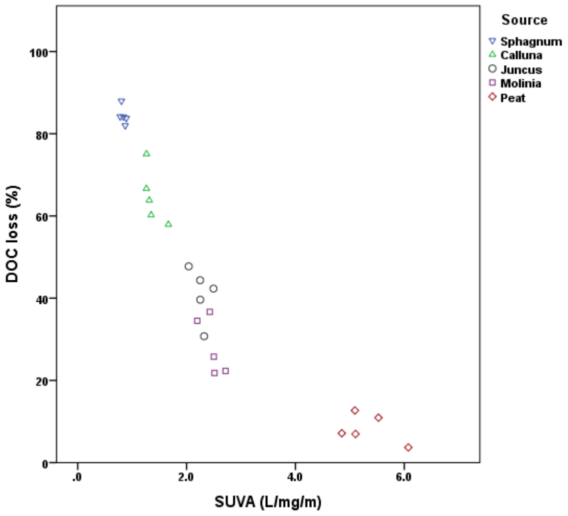

Supplementary Figure 3: Correlation between SUVA, indicating aromaticity of DOC, and % loss during biodegradable DOC incubations for the different sources (Spearman's ρ=-0.978, p<0.001).

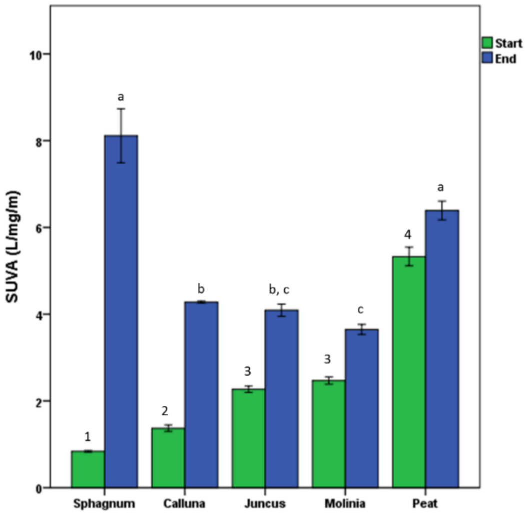

Supplementary Figure 4: SUVA values (indicating aromaticity) for DOC sources before and after BDOC incubations. Numbers indicate statistically similar groupings pre-incubation and letters indicate statistically similar groupings post-incubation. Error bars at one standard error.

Supplementary Table 1: Carbon and nitrogen % and C:N ratios for homogenised starting material, mean of two replicates.

**DOC source**	**%C**	**%N**	**C:N**
Peat soil	56.6	1.8	29.9
Sphagnum	41.5	0.4	93.7
Calluna	51.4	0.9	56.5
Juncus	43.7	1.0	42.1
Molinia	44.0	1.2	35.7

Supplementary Table 2: Results of ANCOVA model for litterbag data (significant terms in the model are highlighted in bold and show the ω^2 estimate of effect size).

	**Vegetation**	**Temperature**	**Water table**
Mass loss	**F=202.26, p<0.001, ω^2=0.927**	F=0.12, p=0.735	F=1.38, p=0.246
Extractable DOC	**F=8.69, p<0.001, ω^2=0.298**	F=3.33, p=0.075	F=2.29, p=0.138
SUVA	**F=27.32, p<0.001, ω^2=0.608**	F=0.82, p=0.370	F=2.70, p=0.108

Supplementary Table 3: literature review of studies comparing peatland vegetation type to DOC concentrations in soils and/or rivers.

Reference	Location	Measurements taken	Findings
Andersson and Nyberg (2009)	18 catchments in Varmland, Sweden	GIS datasets of topography, % wetland and vegetation type compared against measurements of DOC, Al, Fe and Si. Mostly coniferous forests with <5% in dwarf-shrub and mire vegetation classes.	Some correlations between vegetation classes and water quality measures, however % wetland, mean slope and topographic wetness index show best correlations.
Armstrong et al. (2012)	Northern England and northern Scotland	DOC in soil water and in artificial drains from areas dominate by *Calluna*, *Sphagnum*, sedges and *Molinia*.	Soil water concentrations in the order *Calluna*>sedges>*Sphagnum*>*Molinia*. DOC in drains also highest in *Calluna* areas.
Armstrong et al., (2015)	Scotland, UK	Net ecosystem exchange, methane flux, porewater DOC, vegetation biomass, peat properties, water table	Plant functional type has a strong control on net ecosystem exchange and methane flux but not porewater DOC
Fenner et al. (2004)	Laboratory study on peat monoliths from Plynlimon, Wales	^{13}C pulse-chase experiment looking at enrichment in *Sphagnum* moss and DOC in porewater.	Recently photosynthesised carbon from *Sphagnum* adds significantly to DOC
Fenner et al. (2007)	Laboratory study on peat monoliths from Plynlimon, Wales	Three-year treatment of elevated CO_2, measuring vegetation changes and DOC concentrations. Also ^{13}C pulse-chase experiment.	*Sphagnum* cover declines, *Juncus* cover increases. Concurrent increase in above-ground biomass and ^{13}C turnover. Higher DOC concentration and amount of $DO^{13}C$.
Gogo et al. (2010)	La Guette peatland, France	DOC in peat pore-water and characterisation of organic matter below *Sphagnum* vs. *Molinia*.	Higher DOC under *Molinia* but greater amount of organic matter and less decomposition under *Sphagnum*.
Gogo et al. (2012)	La Guette peatland, France	DOC in peat pore-water and in neighbouring streams	Higher peat pore-water concentrations in plots dominated by *Molinia* and *Betula Spp.* than *Sphagnum*. Peaks in September.

Helliwell et al. (2007)	Across the UK	N species and DOC in lakes and reservoirs compared against catchment characteristics	Negative correlation between % grassland and % bare rock and DOC for some sites.
Parry et al. (2015)	119 catchments across the Penines, UK	Riverine [DOC] and UV quality measures. Could not distinguish *Sphagnum* using remote sensing. Classified vegetation as bare peat, ericaceous shrubs, sedges, graminoids and mixed.	Vegetation has control on [DOC] and quality, however slope stronger factor
Peacock et al. (2013)	Afon Ddu catchment, Migneint, Wales	Pool depth, vegetation and DOC concentration of 60 bog pools created by ditch blocking	Vegetation type in pool does not influence DOC concentration, however pools are draining large upstream areas and have short residence times.
van den Berg et al. (2012)	41 sites, mainly northern England but some across UK	Soil porewater DOC over a year. C:N, precipitation, temperature, soil type and vegetation cover as explanatory variables.	DOC higher under moorland vegetation class vs heathland, woodland and grassland. C:N and precipitation main factors in model of DOC.
Vestgarden et al. (2010)	Montane system, south Norway	DOC at 0-10 and 10-20 cm under *Sphagnum*, *Molinia* and *Calluna*. Also soil organic carbon.	*Molinia* and *Calluna* have higher DOC at 10cm and increase in summer/autumn, timing with litter production. *Sphagnum* has stable year-round DOC and is higher than others at 10-20cm due to large C pool.

Supplementary Table 4: Summary temperature and water table data for Dartmoor sites used in litterbag experiments showing the mean and range in brackets.

**Site number (elevation)**	**Temperature at 5 cm (°C)**	**Depth to water table (m)**
2 (365 m asl)	10.25 (19.82 – 0.88)	0.05 (-0.02 – 0.14)
5 (503 m asl)	8.89 (18.51 – 0.16)	0.12 (0.00 – 0.23)
7 (528 m asl)	8.85 (18.13 – 0.66)	0.11 (0.01 – 0.35)

Method for temperature and water table logging in Supplementary Table 4.

Absolute pressure was recorded at 15 minute intervals using a non-vented pressure transducer (HOBO U20-001-04 Water Level Data Logger) suspended from the top of each dip well with plastic-coated stainless steel cable. To compensate the dip well pressure readings for barometric pressure, an additional sensor was placed above-ground at a single location and was set to log at the same interval. Water level was recorded manually from a fixed point at the top of the dip well when the loggers were downloaded and during other site visits. The compensated pressure readings were calibrated to these manual water level measurements to give a continuous time series of water level relative to the bog surface. Temperature was recorded and logged every 5 minutes at a depth of 5 cm below the bog surface using a logger with external temperature sensors (HOBO U23 Pro v2 Temperature Data Logger).

References

Andersson, J.-O., Nyberg, L., 2009. Using official map data on topography, wetlands and vegetation cover for prediction of stream water chemistry in boreal headwater catchments. Hydrol. Earth Syst. Sci. 13, 537–549. doi:10.5194/hess-13-537-2009

Armstrong, A., Holden, J., Luxton, K., Quinton, J.N., 2012. Multi-scale relationship between peatland vegetation type and dissolved organic carbon concentration. Ecol. Eng. 47, 182–188. doi:10.1016/j.ecoleng.2012.06.027

Armstrong, A., Waldron, S., Ostle, N.J., Richardson, H., Whitaker, J., 2015. Biotic and Abiotic Factors Interact to Regulate Northern Peatland Carbon Cycling. Ecosystems 18, 1395–1409. doi:10.1007/s10021-015-9907-4

Fenner, N., Ostle, N., Freeman, C., Sleep, D., Reynolds, B., 2004. Peatland carbon efflux partitioning reveals that Sphagnum photosynthate contributes to the DOC pool. Plant Soil 345–354.

Fenner, N., Ostle, N.J., McNamara, N., Sparks, T., Harmens, H., Reynolds, B., Freeman, C., 2007. Elevated CO2 Effects on Peatland Plant Community Carbon Dynamics and DOC Production. Ecosystems 10, 635–647. doi:10.1007/s10021-007-9051-x

Gogo, S., Albéric, P., Laggoun-Défarge, F., Binet, S., Aurouet, a., 2012. Spatial and temporal variations of dissolved organic carbon and inorganic carbon concentrations and δ^{13}C in a

peatland-stream continuum: implications of peatland invasion by vascular plants. Biogeosciences Discuss. 9, 3515–3544. doi:10.5194/bgd-9-3515-2012

Gogo, S., Laggoun-Défarge, F., Delarue, F., Lottier, N., 2010. Invasion of a Sphagnum-peatland by Betula spp and Molinia caerulea impacts organic matter biochemistry. Implications for carbon and nutrient cycling. Biogeochemistry 106, 53–69. doi:10.1007/s10533-010-9433-6

Helliwell, R.C., Coull, M.C., Davies, J.J.L., Evans, C.D., Norris, D., Ferrier, R.C., Jenkins, a., Reynolds, B., 2007. The role of catchment characteristics in determining surface water nitrogen in four upland regions in the UK. Hydrol. Earth Syst. Sci. 11, 356–371. doi:10.5194/hess-11-356-2007

Parry, L.E., Chapman, P.J., Palmer, S.M., Wallage, Z.E., Wynne, H., Holden, J., 2015. The influence of slope and peatland vegetation type on riverine dissolved organic carbon and water colour at different scales. Sci. Total Environ. 527-528, 530–539. doi:10.1016/j.scitotenv.2015.03.036

Peacock, M., Evans, C.D., Fenner, N., Freeman, C., 2013. Natural revegetation of bog pools after peatland restoration involving ditch blocking—The influence of pool depth and implications for carbon cycling. Ecol. Eng. 57, 297–301. doi:10.1016/j.ecoleng.2013.04.055

van den Berg, L.J.L., Shotbolt, L., Ashmore, M.R., 2012. Dissolved organic carbon (DOC) concentrations in UK soils and the influence of soil, vegetation type and seasonality. Sci. Total Environ. 427-428, 269–76. doi:10.1016/j.scitotenv.2012.03.069

Vestgarden, L.S., Austnes, K., Strand, L.T., 2010. Vegetation control on DOC, DON and DIN concentrations in soil water from a montane system, southern Norway. Boreal Environ. Res. 15, 565–578.
